# Mueller matrix polarization parameters correlate with local recurrence in patients with stage III colorectal cancer

**DOI:** 10.1038/s41598-023-40480-3

**Published:** 2023-08-17

**Authors:** Kseniia Tumanova, Stefano Serra, Anamitra Majumdar, Jigar Lad, Fayez Quereshy, Mohammadali Khorasani, Alex Vitkin

**Affiliations:** 1 Department of Medical Biophysics, University of Toronto, Toronto, Canada; 2 Department of Laboratory Medicine and Pathobiology, University of Toronto, Toronto, Canada; 3 Department of Surgery, University of Toronto, Toronto, Canada; 4 Department of Surgery, University of British Columbia, Victoria, Canada; 5 Division of Biophysics and Bioimaging, Princess Margaret Cancer Centre, University Health Network, Toronto, Canada; 6 Department of Radiation Oncology, University of Toronto, Toronto, Canada

**Keywords:** Biophysics, Cancer, Biomarkers, Optics and photonics

## Abstract

The peri-tumoural stroma has been explored as a useful source of prognostic information in colorectal cancer. Using Mueller matrix (MM) polarized light microscopy for quantification of unstained histology slides, the current study assesses the prognostic potential of polarimetric characteristics of peri-tumoural collagenous stroma architecture in 38 human stage III colorectal cancer (CRC) patient samples. Specifically, Mueller matrix transformation and polar decomposition parameters were tested for association with 5-year patient local recurrence outcomes. The results show that some of these polarimetric parameters were significantly different (*p* value < 0.05) for the recurrence versus the no-recurrence patient cohorts (Mann–Whitney U test). MM parameters may thus be prognostically valuable towards improving clinical management/treatment stratification in CRC patients.

## Introduction

Colorectal cancer is one of the most common malignancies. Every year an estimated 1.4 million people worldwide are diagnosed with colorectal carcinoma (CRC)^1,2^. Although surgical resection is the main treatment at present, the significant incidence of tumour recurrence following the procedure is strongly linked to a diminished chance of survival^3^. Local recurrence (LR) plays a critical role in determining the outcome of patients who have undergone surgery for CRC^3^; LR refers to the regrowth of cancer cells in the same location where the tumour was initially removed^3^. Generally, the LR rate of colon cancer treated for cure is thought to be lower than that of rectal cancer; reported rates are below 10% for the former and 5–19% for the latter, depending on the stage and other clinical variables^4–6^. The ability to predict which patients are likely to fail due to LR could be useful for creating a personalized and more patient-centred treatment plan. For example, such patients may be considered for adjuvant radiation or chemotherapy and may also be suitable for more intensive follow-up after curative resection^3^.

Few biomarkers based on genetic analysis of the cellular DNA (e.g., OncotypeDx, ColoPrint, ColoGuideEx, ColoGuidePro), for predicting distant recurrence in Stage II and III CRC, are currently being investigated for clinical workup^7–11^. Despite being promising for recurrence prediction, the search for alternative sources of prognostic information is necessitated by numerous studies demonstrating the various shortcomings of most of these tests, as well as their high cost^10,11^. Cellular compartment aside, increasing evidence supports the prognostic value of the tumour microenvironment, such as tumour stromal architecture^12–16^, particularly desmoplastic response (DR)^17–20^. DR is linked to the growth and structural reorganization of collagenous fibres in the most invasive tumour front regions. In order to evaluate DR, stromal maturity is divided into three categories (immature, intermediate, and mature); recent studies show that stromal maturity is correlated with 5-year relapse-free survival and locoregional recurrence^12,13^. Nonetheless, despite its promise, assessment (either qualitative or quantitative) of DR is hampered by inter-observer variability and analysis subjectivity, which makes its clinical implementation challenging.

Towards quantifying DR and beyond, collagen can be visualized using a variety of optical techniques. Scanning and transmission electron microscopies offer high-resolution visualization of individual collagen fibres but are expensive, involve complicated specimen preparation, and provide nanoscale detail that is excessive for many applications^21^. Second harmonic generation (SHG) imaging is sensitive, specific for fibrillar collagen, and amenable to quantification methods but lengthy imaging times, limited fields of view, high cost and technical complexity of its advanced microscope components hinder its clinical use^22,23^. Another popular method to image fibrillar stroma is staining a histology slide with picrosirius red or Mason Trichrome and imaging with a microscope^24^. However, utility of this method has been hindered by the multi-step sample preparation and reproducibility issues caused by differences in staining procedures^25^.

In contrast, polarized light microscopy (PLM) offers a comparatively simple, rapid, and robust approach to address these disadvantages. PLM provides strong stromal contrast and wide fields of view with minimal sample preparation. Several recent reviews of polarimetry have summarized its potential in biomedicine, highlighting its applications in different cancer types such as breast, cervix, prostate, brain, and colon^26–28^. Among the extensively investigated techniques, the LC-PolScope and polychromatic polarization microscopy (PPM) stand out due to their cost-effectiveness and high resolution advantages^29–33^. Significant contributions in this field have been made by Keikhosravi et al. who successfully quantified collagen organization in histopathology samples using liquid crystal-based polarization microscopy and real-time polarization microscopy of fibrillar collagen, respectively^31,32^. However, it is important to consider their limitations. The LC-PolScope relies on liquid crystal devices, which require frequent calibration due to component variations^30^. Additionally, both LC-PolScope and PPM primarily focus on imaging birefringent structures to acquire specimen retardation and its principal axis orientation, both useful metrics for specific applications. Nevertheless, in complex biological tissues, a polarization technique capable of providing a more comprehensive understanding of various structural tissue reorganizations may be advantageous^29,30^.

A novel variant of cross-polarized light microscopy, recently proposed by our group^34–37^ and combined with unsupervised clustering algorithm models, assessed the prognostic value of collagenous stroma to predict 5-year patient survival and showed initial promise^34^. However, no correlation was found with LR outcomes, another clinically relevant endpoint. Hence an expanded and more powerful polarimetric technique that is sensitive to microstructural changes must be developed for obtaining this clinically relevant information on biomedical specimens. In the current paper, we address this challenge and demonstrate that Mueller matrix (MM) polarimetry imaging may provide useful contrasts to stratify patients into LR-correlated groups, further suggesting that tumour stroma may indeed contain information of significant prognostic value. This work describes the extraction of MM polarimetric parameters that may have explicit associations with specific microstructural/biophysical features of CRC tissues, and statistical analysis to explore correlations with actual clinical outcomes (5-year LR). The results suggest that this quantitative methodology can separate outcome groups, warranting further study and validation towards a simple and robust tool for incorporating stromal-based prognosis into potential clinical use.

## Methods

### Ethics

Institutional ethics approval was obtained from the University Health Network (Toronto, Ontario, Canada). The need for patients’ consent was waived by the ethics board due to the retrospective nature of the study, anonymization of personal health information, and the results of H&E analysis having already been discussed with patients. All procedures and handling of patient data were conducted in accordance with the University Health Network Research Ethics Board guidelines/approvals.

### Cancerous tissue samples

**Figure 1:**
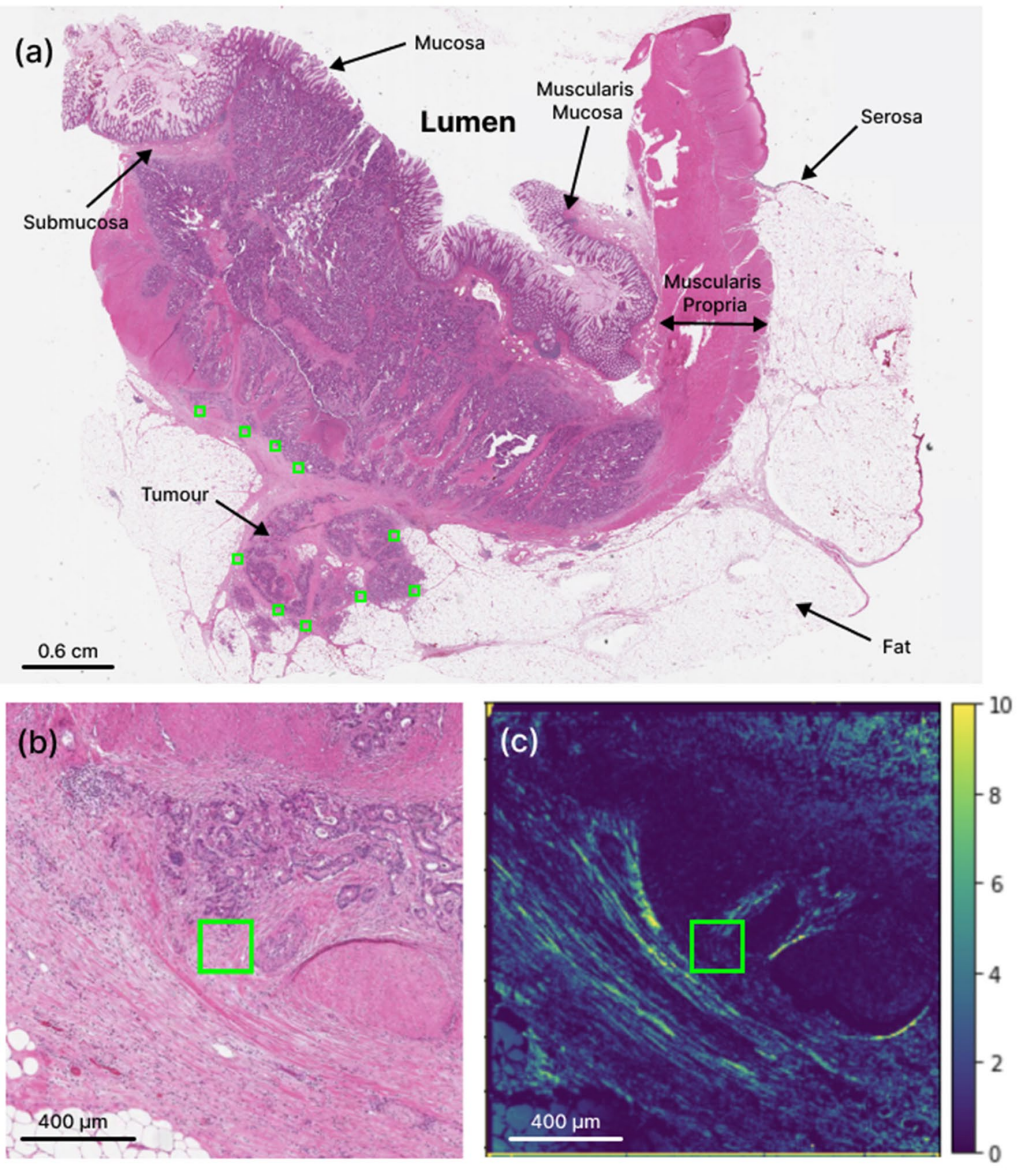
Whole-slide histologic and polarimetric imaging of a stage III CRC sample for qualitative ROI-based analysis. (**a**) Overview image of H&E-stained slide, showing different CR tissue types and pathologist-selected ROIs (green squares at the leading edge of the tumour). Zoomed-in (**b**) H&E and (**c**) retardance (see Section “Polarimetric method”) images of the region around the left-most ROI in (**a**). The brighter areas in (**c**) represent birefringent tissues that contain more collagenous stroma.

This study used 38 archival surgical resection samples of Stage III left-sided colorectal cancer patients prior to receiving adjuvant chemotherapy. Clinical data (including clinical outcomes such as 5-year survival, 5-year local recurrence, etc.) were available to assess correlations. The no-LR cohort contained 29 patients; consequently 9 patients belonged to the local recurrence group. The analysis used unstained 4.5 µm thick sections on charged microscope slides, from formalin-fixed and paraffin-embedded blocks. Minimal sample preparation involved chemical dewaxing to avoid possible polarization imaging artifacts^34–37^. No further processing was required for polarimetric imaging. Adjacent slides were H&E stained and scanned at 20X magnification on an Aperio ScanScope CS (Leica Biosystems, USA) for the pathologists’ region-of-interest (ROI) selection (Fig. 1a).

### ROI selection and histology

In this study, the most representative section of each patient's tissue resection was selected based on the depth of tumour invasion. To ensure systematically random sampling within the peri-tumoural region, an experienced gastrointestinal pathologist (SS) employed a “grid rule” approach^38^. The pathologist used an overlaying grid within the peri-tumoural area, identifying regions of interest (ROIs) measuring approximately 200 μm × 200 μm on each slide. The sampling grid allowed for an even distribution and equal-sized ROIs within the peri-tumoural region, minimizing potential biases in the selection process. The ROIs were marked at the interface of the invasive tumour front and stroma, excluding cancer cells when possible (Fig. 1a). Microscopically evident structures such as smooth muscle tissue, lymphoid follicles, and large vessels were excluded as they are considered part of the native constituents of the large bowel and are likely not relevant to the analysis. The number of ROIs per patient slide ranged from 3 to 14 (356 ROIs in total over the 38 patients), depending on the size of the tumour and morphological characteristics of the stroma at the invasive tumour front. To avoid selection bias, the pathologist was blinded to the polarimetry images and clinical outcome data, such as 5-year local recurrence. The size of the ROI was carefully chosen to strike a balance between ensuring robust statistical analysis and capturing the inherent heterogeneity of the stromal region, while also maintaining adequate spatial resolution as reported in other studies^35,36^. This approach aimed to optimize the selection of the ROI size, taking into consideration the need for reliable statistics and the diverse characteristics of the stroma. Visual tissue landmarks were used to transfer these ROIs from the H&E images onto the adjacent unstained slides imaged polarimetrically (Fig. 1b, c). Image processing and polarimetric analysis were then performed using Python programming language.

### Polarimetric method

**Figure 2:**
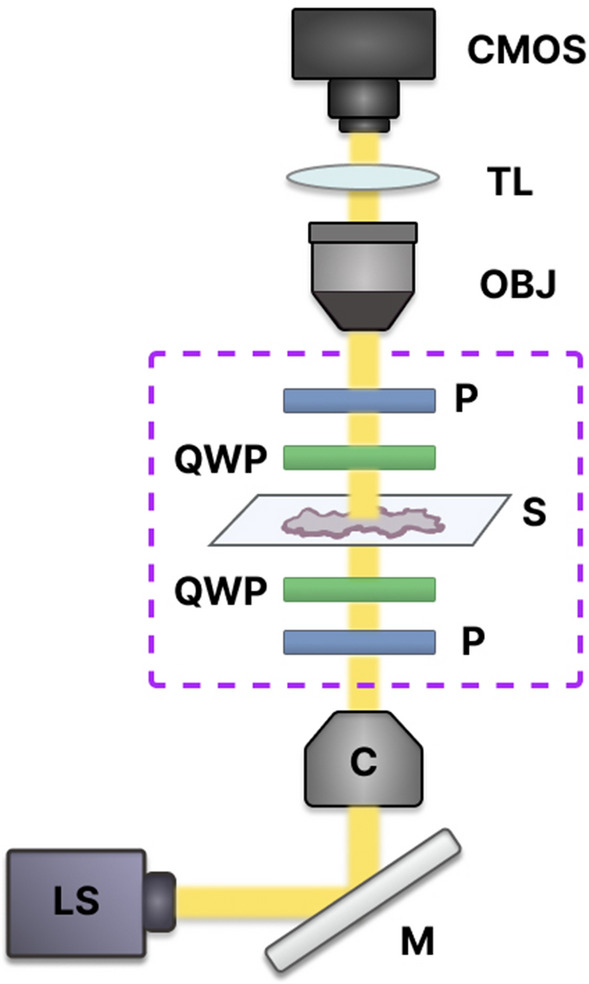
Experimental setup for Muller matrix microscopy. Light enters from below the sample (S), passes through a collimator (C) and polarization state generator (PSG), consisting of a linear polarizer (P) and a quarter-wave plate (QWP). After interacting with the sample, light passes through a polarization state analyzer (PSA), consisting of QWP and P. Finally, light is collected by the microscope objective lens (OBJ) and an image is captured by a CMOS camera. LS, light source; M, mirror; TL, tube lens. The polarimetry module is outlined with a dotted rectangle.

The multiscale Mueller polarimetry module for a stereo zoom microscope (Axio Zoom V16, Zeiss with the objective lens Plan Neofluar Z 1X/0.25 NA), recently designed by our group, was used for this study^39^. Briefly, the polarization of the incident light is modulated by a Polarization State Generator (PSG) comprising a rotatable linear polarizer (LPVISE100-A, Thorlabs) followed by a rotatable quarter wave plate (QWP) (AQWP05M-600, Thorlabs). The unstained tissue sample is viewed through suitable imaging optics including a Polarization State Analyser (PSA) made of the same elements as the PSG, but in reverse order (Fig. 2). The specific arrangement and orientations of the QWPs help in generating and analyzing the circular polarization components of the light, providing valuable additional information (over and above the linear polarization states and interactions) about the optical properties and structural characteristics of the sample. The microscope LED [Illuminator HXP 200C (D), Zeiss] is a 310 W uncollimated white light source passing through a 630 nm filter (ET630/75 or ZET630/10, Chroma). This is a reasonable tissue-optics wavelength choice because tissue scattering and hemoglobin absorption are relatively low at longer visible wavelengths^40^. The camera was the ORCA-Flash4.0 V3 Digital CMOS by Hamamatsu. Its pixels are 6.5 × 6.5 μm^2^, arranged in a 2048 × 2048 array. Microscope optics yielded lateral resolution of < 2.2 μm. The field of view in this study was 1.66 × 1.66 mm^2^, corresponding to an effective magnification of 8X. Taking this effective magnification into account, we calculated the pixel size to be 0.81 × 0.81 μm^2^.

To extract more information from the polarimetry images, the methodology presented here expands on our previous rotating-crossed-polarizers approach^34–37^ towards a full MM analysis. The Mueller matrix is a sample transfer function that describes how the polarization of light changes due to its interactions with the sample, and thus represents the polarization properties of tissue. These properties are contained in its Mueller matrix M that links the input and output light states through a simple linear relationship:

1 $$\begin{array}{*{20}c} {S_{out} = MS_{in} } \\ \end{array}$$

where ${S}_{in}$ is the input 4-element Stokes vector (input polarization state) and ${S}_{out}$ is the output 4-element Stokes vector (output polarization state). The Mueller matrix thus contains 16 parameters and can be written as:

2 $$\begin{array}{*{20}c} {M = \begin{array}{*{20}c} {m_{11} } & {m_{12} } & {\begin{array}{*{20}c} {m_{13} } & {m_{14} } \\ \end{array} } \\ {m_{21} } & {m_{22} } & {\begin{array}{*{20}c} {m_{23} } & {m_{24} } \\ \end{array} } \\ {\begin{array}{*{20}c} {m_{31} } \\ {m_{41} } \\ \end{array} } & {\begin{array}{*{20}c} {m_{32} } \\ {m_{42} } \\ \end{array} } & {\begin{array}{*{20}c} {\begin{array}{*{20}c} {m_{33} } & {m_{34} } \\ \end{array} } \\ {\begin{array}{*{20}c} {m_{43} } & {m_{44} } \\ \end{array} } \\ \end{array} } \\ \end{array} } \\ \end{array}$$

where ${m}_{ij}$ are the elements of Mueller matrix.

Despite the seeming simplicity of Eqs. (1) and (2), the matrix algebra for any realistic measurement gets complicated very quickly; more to the point, the derived MM of tissue contains many “numbers” (16 elements!) of uncertain biophysical meaning and significance. The challenges of MM polarimetry are thus (A) to measure MM accurately, and (B) to extract meaningful biophysical metrics from it. To address (A), we used 24 direct Stokes vector images rather than the minimum of 16 to improve robustness and SNR^39,41^. The setup was calibrated by measuring the Mueller matrices of standard samples, including air and retarders. Calibration is performed pixel-by-pixel to reduce spatially dependent distortions, such as those caused by off-axis oblique rays. The reconstructed polarimetry images have slightly lower resolution compared to a single transmission Stokes image due to slight image shifts resulting from different setting of PSG and PSA. This rotation induces minor angular variations in optical component thickness and/or optical properties, which cause slight shifts in image location and potential loss of focus^39,42^.

To address potential artifacts in image alignment, we used pyStackReg, a Python library commonly employed for image co-registration^43^. This method incorporates bilinear interpolation, which calculates pixel values based on the weighted average of neighboring pixels, facilitating smooth transitions during image alignment. Although this can introduce slight variations in pixel values and potentially smooth out fine details or cause minor blurring in registered images, we performed a rigorous validation process to ensure the reliability of our analysis. Through comprehensive comparisons between interpolated and non-interpolated images, we consistently observed similar trends and patterns, suggesting that the interpolation did not introduce substantial distortions or biases that would affect the validity of our results.

To address (B), we (i) decomposed the MM using Lu–Chipman polar decomposition (MMPD) followed by selected metrics extraction^44^, and (ii) calculated Mueller matrix transformation (MMT) parameters^45^. There are many other possible polarimetric measures and decomposition techniques to choose from; these two methods were chosen to demonstrate proof of concept due to their relative simplicity and potential biophysical interpretation of the derived numbers^28^.

(i) MMPD represents any physically realistic MM as the product of depolarizer (${\mathrm{M}}_{\Delta }$), retarder (${\mathrm{M}}_{\mathrm{R}}$), and diattenuator (${\mathrm{M}}_{\mathrm{D}}$) matrices:
  3 $$\begin{array}{*{20}c} {M = {\text{M}}_{\Delta } {\text{M}}_{{\text{R}}} {\text{M}}_{{\text{D}}} } \\ \end{array}$$

As matrix multiplication is not commutative (order matters), the above decomposition order is not unique^46^. Several research studies have tackled this issue, including offering alternate decomposition algorithms^47–50^. Nevertheless, the Lu-Chipman approach seems to work adequately in biological media^26,28^. From Eq. (3), MMPD parameters $\Delta$, R, and D can then be calculated, representing depolarization, retardance, and diattenuation properties of the tissue sample, respectively:

4a $$\begin{array}{*{20}c} {\Delta = 1 - \frac{{\left| {{\text{tr}}\left( {{\text{M}}_{\Delta } } \right) - 1} \right|}}{3}} \\ \end{array}$$

4b $$\begin{array}{*{20}c} {{\text{R}} = \cos^{ - 1} \left[ {\frac{{{\text{tr}}\left( {{\text{M}}_{{\text{R}}} } \right)}}{2} - 1} \right]} \\ \end{array}$$

4c $$\begin{array}{*{20}c} {{\text{D}} = \frac{1}{{{\text{m}}_{11} }}\sqrt {{\text{m}}_{12}^{2} + {\text{ m}}_{13}^{2} + {\text{m}}_{14}^{2} } } \\ \end{array}$$

Further, the retardance R is composed of linear and circular contributions^26^. In this study, we analyze the linear retardance and circular retardance of CRC samples via Eqs. (5a) and (5b), respectively:

5a $$\begin{array}{*{20}c} {\delta = \cos^{ - 1} \left\{ {\sqrt {\left[ {M_{R} \left( {2,2} \right) + M_{R} \left( {3,3} \right)} \right]^{2} + \left[ {M_{R} \left( {3,2} \right) - M_{R} \left( {2,3} \right)} \right]^{2} } - 1} \right\}} \\ \end{array}$$

5b $$\begin{array}{*{20}c} {\Psi = \tan^{ - 1} \left\{ {\frac{{M_{R} \left( {3,2} \right) - M_{R} \left( {2,3} \right)}}{{M_{R} \left( {2,2} \right) + M_{R} \left( {3,3} \right)}}} \right\}} \\ \end{array}$$

(ii) Instead of matrix manipulations as pursued via MMPD or other decomposition approaches, He et al.have proposed MMT method by fitting the original MM elements to the trigonometric functions in polar coordinates to obtain a set of parameters for quantitatively describing the characteristics of anisotropic scattering media^45^:
  6a $$\begin{array}{*{20}c} {{\text{t}}_{1} = \frac{{\sqrt {\left( {m_{22} - m_{33} } \right)^{2} + \left( {m_{23} + m_{32} } \right)^{2} } }}{2}} \\ \end{array}$$
  6b $$\begin{array}{*{20}c} {{\text{b}} = \frac{{m_{22} + m_{33} }}{2}} \\ \end{array}$$
  6c $$\begin{array}{*{20}c} {{\text{P}}_{{\text{L}}} = \sqrt {m_{21}^{2} + m_{31}^{2} } } \\ \end{array}$$
  6d $$\begin{array}{*{20}c} {{\text{q}}_{{\text{L}}} = \sqrt {m_{42}^{2} + m_{43}^{2} } } \\ \end{array}$$
  6e $$\begin{array}{*{20}c} {\beta = \frac{{\left| {m_{23} - m_{32} } \right|}}{2}} \\ \end{array}$$

Previous efforts to assign biophysical meaning to these MMT and to the above-discussed MM decomposition parameters have suggested the following: (a) the MMPD parameter ∆ and the MMT parameter b are sensitive to the sample depolarization properties^51,52^; (b) the MMPD parameter D and the MMT parameters t_1_ and P_L_ are sensitive to the sample’s anisotropy^52,53^; (c) the MMPD parameter δ and the MMT parameter q_L_ can reveal the alignment and density of the fibrous structures due to their birefringence^26,52,53^; (d) retardance-related information (e.g., R, δ, and $\Psi$ in MMPD’s Eqs. 4b, 5a, and 5b) is mainly contained in the sub-matrix [ m_22_ m_23_ m_24_; m_32_ m_33_ m_34_; m_42_ m_43_ m_44_]^26,28^, as also reflected in the MMT parameter q_L_ (the root-mean-square of m_42_ and m_43_ is one of the rotation-invariant parameters representing the ability of transforming between linear and circular polarizations^52,53^); (e) the magnitude of the difference between m_23_ and m_32_ ($\upbeta$ in Eq. 6e) gives a measure of optical rotation (circular birefringence) and coexistence of multiple anisotropic effects in the medium^28,54,55^. Similarly, circular retardance $\Psi$ has been linked to the presence of chiral molecules (e.g., sugars, DNA, enzymes), the changes of structures and/or concentrations of which are recognized as factors contributing to cancer development^26,56–58^. For a more comprehensive understanding, please refer to Table 1, which demonstrates the relationship between these polarimetric parameters and their potential biophysical meaning.

**Table 1:** Examined polarization metrics and their potential biophysical meaning for characterizing biological tissue properties.

Polarimetric parameters	Biophysical meaning
$\Delta$	Heterogenous nature of biological tissue
$b$	Spatial distribution of sub-wavelength “small” cellular organelles
$D, {t}_{1}$, ${P}_{L}$	Directional heterogeneity of fibrous structures
$\mathrm{R}, \delta$, ${q}_{L}$	Alignment and density of collagenous fibers
$\Psi$	Presence of chiral molecules
$\beta$	Presence of chiral molecules and/or arrangement of collagen fibers

Mueller matrix elements are excluded due to their complexity in interpretation within the complex and heterogeneous nature of biological tissue.

Clearly then, there are some potential metrics candidates in the analysis/interpretation of MM (10 metrics reported in Table 1 and 9 MM elements listed above), and their optimum choice will likely be *task-specific*. That is, MM metrics that potentially delineate pathologic from normal tissue will likely be (at least in part) different from those that may correlate with 5-year disease-free survival or with probability of local recurrence. With that in mind, we tested the various MM polarization metrics mentioned above for possible correlations with local recurrence in a clinical cohort of 38 Stage-III CRC patients. The results below summarize our findings for the most significant “hits”.

### Statistical method

The MM elements were computed individually for every pixel, yielding 62,500 values for each ROI. To aggregate the pixel-level data to the patient level, a two-step averaging method was applied. First, the median value for the MM elements across all pixels was calculated for each ROI. Then, the mean value was computed across the 3–14 ROIs within a given patient sample. The resulting Mueller matrix was used to derive a set of 19 polarimetric parameters for each patient. To compare these parameters between the patient outcome groups (LR vs. No LR), we first performed the Shapiro–Wilk test to check the normality assumption of the data; analysis indicated that the data was not normally distributed. Therefore, we used the Mann–Whitney (MW) U test to assess the null hypothesis that the categorical groups came from the same distribution^59^. The MW U test is a non-parametric or 'distribution-free’ test that is appropriate for non-normally distributed data. A statistically significant result (*p* < 0.05) would indicate that there are significant differences between the groups, implying that not all samples were drawn from the same distribution.

It is important to note that no extreme outliers were identified during the data analysis process. The dataset exhibited a consistent pattern without any values that deviated significantly from the overall trends. Therefore, there was no need to exclude any data points from the analysis, and all the available data were utilized for the statistical comparisons between patient outcome groups (as reported in Table 2).

**Table 2:** Resultant statistics for the examined polarization metrics.

	D		${\mathrm{t}}_{1}$		${\mathrm{q}}_{\mathrm{L}}$		|${\mathrm{m}}_{43}$|		$\beta$	
	No LR	LR	No LR	LR	No LR	LR	No LR	LR	No LR	LR
µ	0.02	0.05	0.005	0.012	0.010	0.03	0.007	0.025	0.005	0.007
$\sigma$	0.01	0.03	0.004	0.011	0.009	0.02	0.007	0.025	0.009	0.005
Median	0.02	0.04	0.004	0.008	0.008	0.01	0.005	0.008	0.002	0.005
p	0.0101		0.0018		0.0031		0.0237		0.0212	

µ represents the mean, and $\upsigma$ is the standard deviation; *p* value < 0.05 indicates a statistically significant difference between the No LR (n = 29) and LR (n = 9) groups. Total of 356 ROIs from 38 patient samples were analyzed.

## Results and discussion

**Figure 3:**
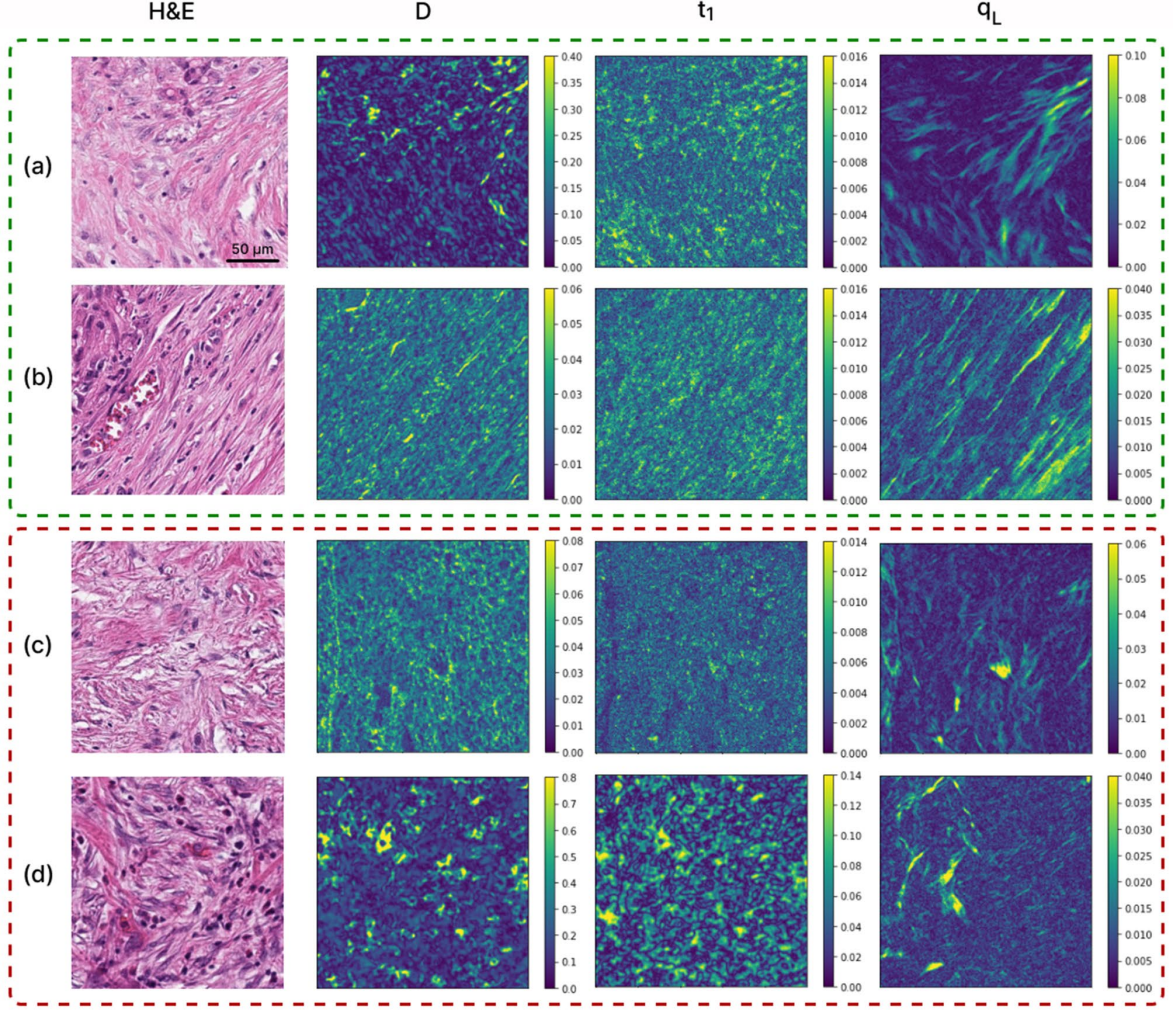
Mueller matrix polarimetry of human stage III left-sided colorectal cancer. H&E, D, t_1_ and q_L_ images of a particular ROI; the former two columns report on tissue anisotropy, whereas the latter right-most column is sensitive to density and alignment of birefringent structures such as collagen. (**a**, **b**) two representative ROIs from a no-local-recurrence patient (green dashed contour). (**c**, **d**) two ROIs from a patient whose colorectal cancer did recur locally within 5 years (red dashed contour). Owing to the highly heterogeneous nature of gastrointestinal tissues structures in general, and CRCs in particular (both intra- and inter-patient), no obvious visually discernable patterns are evident. Quantification followed by statistical correlative analysis is required; for details, see text.

Figure 3 displays the images of derived MM parameters D, t_1_ and q_L_, and for patients with different LR outcomes. Two representative ROIs were selected on each slide to illustrate intra- and inter-patient heterogeneity of CRC. As seen, besides some tentative qualitative visual differences, it is challenging to determine whether local recurrence status is reflected in these various polarization images. Obviously quantitative analysis is required, consisting of calculating various summary polarization metrics for each ROI, averaging across all ROIs for each patient, and checking the results for correlation with LR status using statistical comparisons as previously described. Of the nineteen examined polarization metrics described above, five showed statistically significant correlations with 5-year local recurrence status. We present these results in three categories: anisotropy-related parameters, retardance-related parameters, and optical activity. The associated statistics for these are summarized in Table 2.

**Figure 4:**
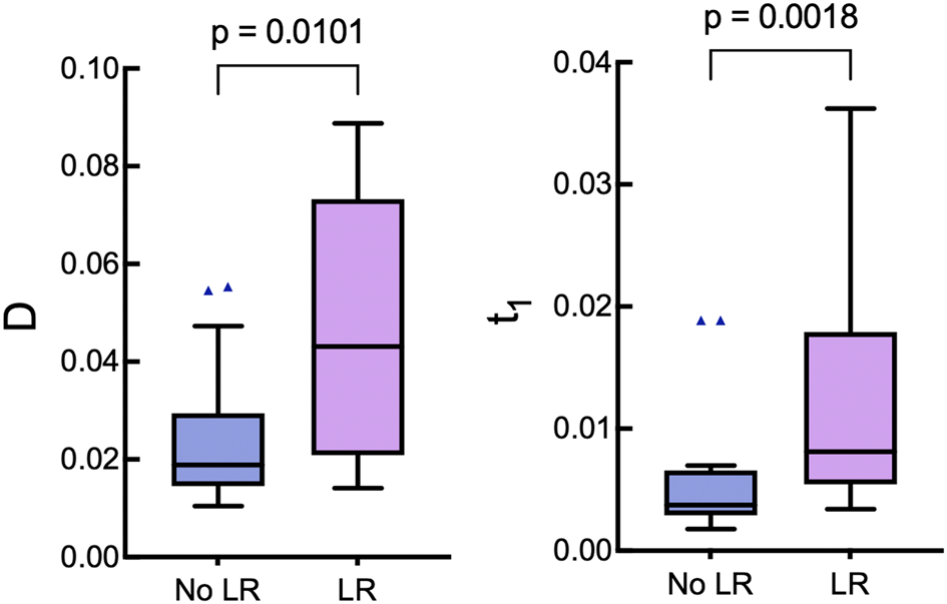
Boxplots showing the comparisons of anisotropy-related metrics D and t_1_ for the two local recurrence outcome groups. For each group, the central black line shows the median, the box indicates the 1st and 3rd quartiles, the whiskers indicate the minimum and maximum values, and outliers are shown with triangles (here and elsewhere, these points were included in the quantitative analysis summarized in Table 1); *p* value < 0.05 indicates a statistically significant difference.

Figure 4 shows the anisotropy-related parameters diattenuation D and degree of anisotropy t_1_, showing statistically significant differences (*p* = 0.0101 and *p* = 0.0018, respectively) in these metrics depending on the patient’s LR status. The patient cohort that went on to recur exhibits greater stromal anisotropy, as indicated by the larger values of both diattenuation and degree of anisotropy. Also noted is the larger spread in the results for the LR group across both metrics; whether this is indicative of greater biological heterogeneity of stage III CRCs that go on to recur, or is caused by the smaller sample size of this cohort (n = 9 vs. n = 29 for the no-LR group) is currently unclear.

**Figure 5:**
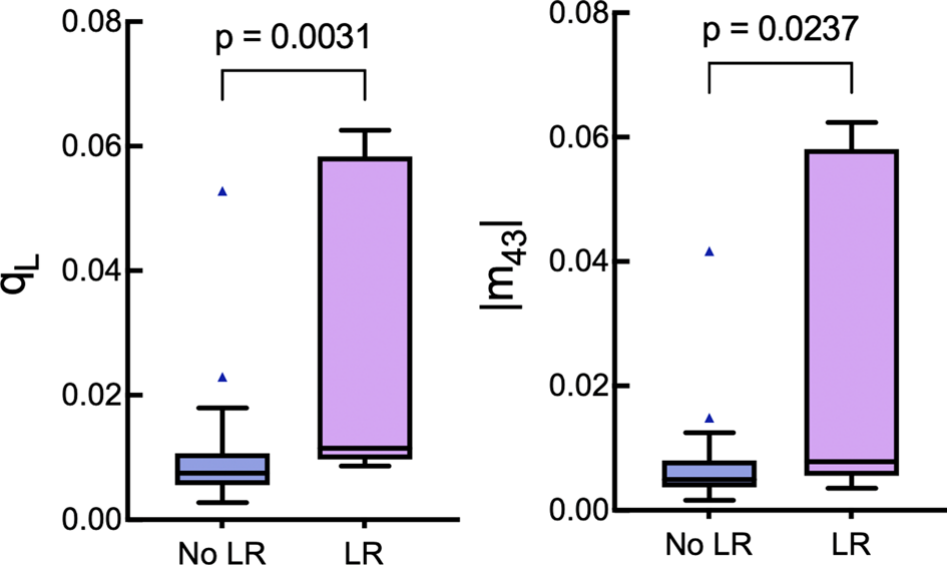
Boxplots showing the comparisons of the retardance-related parameters q_L_ and |m_43_| for the two local recurrence outcome groups. See Fig. 4 caption for explanation.

Figure 5 displays a similar summary for the retardance-related parameters, linear-to-circular polarization conversion ${\mathrm{q}}_{\mathrm{L}}$ (*p* = 0.0031) and the absolute value of the Mueller matrix element m_43_ (*p* = 0.0237). Our findings show that patients who experienced local CRC relapse had significantly higher mean values (and larger spread) of both parameters than those who did not. Unlike these two, and thus somewhat surprisingly, the other often reported retardance parameter—retardance R of Eq. (4b) derived from polar decomposition—did not show significant association with LR status (*p* = 0.0526). However, considering its proximity to statistical significance, this observation may suggest the influence of the limited dataset size. Gathering more data points could potentially lead to reaching statistical significance in the future.

While the actual mechanism of local CRC relapse remains unknown, alterations in collagen structure in the peri-tumoural region may provide a potential explanation of the trends shown in Figs. 4 and 5. Collagen is the major component of the extracellular matrix surrounding the tumour, and in fact tumour-associated collagen signatures (TACS) have been proposed as biophysical metrics of tumour growth, development, and invasion^60^. Specifically in CRC, high-density type I collagen has been associated with poor prognosis in colon carcinoma^61^. Furthermore, type I collagen-rich environments have been shown to induce mesenchymal gene expression and invasion^62^. Increased collagen fiber alignment and stiffness have also been linked to tumourigenesis in colon carcinoma tissues^62^. Our findings suggest that the structural reorganization of collagen fibers is reflected in higher values of anisotropy- and retardance-related parameters in the CRC patients whose cancers recurred. Thus, Mueller matrix polarimetry could be a valuable tool for the non-invasive assessment of collagen fiber structure in CRC and the identification of patients at high risk of local recurrence.

**Figure 6:**
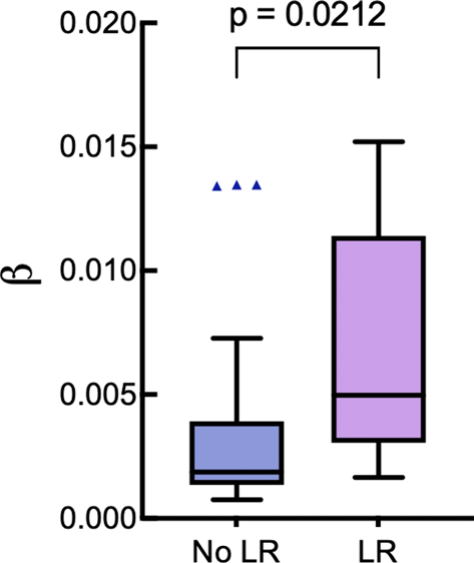
Boxplot showing the comparisons of the circular birefringence parameter β for the two local recurrence outcome groups. See Fig. 4 caption for explanation.

Finally, Fig. 6 shows a statistically significant difference between the LR-related groups for the parameter β (*p* = 0.0212). As mentioned, this parameter reflects optical activity, the ability of certain materials to rotate the plane of linearly polarized light. In the peri-tumoural region, circular birefringence parameters provide insights into the presence of asymmetric optically active chiral molecules (glucose, proteins, lipids) and changes in collagen fiber architecture^26,28^. However, no significant difference is observed in circular retardance parameter $\Psi$ between the LR and LR-free groups (*p* = 0.5845), suggesting that molecular asymmetry is not the main factor driving the observed variations. Instead, the density, orientation, and interaction of collagen fibers with other tissue components might contribute to the overall polarization changes, indicating the presence of multiple anisotropic effects^26–28^.

Moving forward, we are actively exploring alternative methods to extract more information from the polarimetric images, with the goal of enhancing quantitative differences between different clinical-outcomes groups. One avenue we are currently investigating is the potential application of supervised machine learning algorithms. By leveraging these algorithms in our polarimetric analysis, there is the potential for identifying patterns that could contribute to predicting patient LR outcomes in the future. However, it is important to note that at this stage, we are not directly predicting patient outcomes, but rather laying the groundwork for potential advancement in this area. With the inclusion of a larger sample size, this may provide valuable insights towards the discovery of independent prognostic biomarkers. We are also investigating the utility of additional MM-derived parameters and pixel intensity distribution features, including higher orders of the central moment (second order statistics) such as skewness and kurtosis^63^. Another thrust is the development of automatic ROI selection to make our method more objective, as the subjectivity of pathologist's ROI selections can potentially affect the results and reduce the robustness of the approach. Finally, the data reduction/representation/averaging of MM results—the transition from pixel-level findings to ROI-level averages and then to patient-level metrics—can be done in several ways (one possible route pursued here); this needs to be investigated and optimized in greater detail. These methodological refinements will be crucial in testing the ability of polarimetric parameters to predict clinical outcomes and address other clinically important questions.

## Conclusion

The stromal collagen within the tumour microenvironment has been extensively studied and has demonstrated promising potential as a prognostic biomarker in colorectal cancer. However, its widespread clinical adoption is hindered by various obstacles such as CRC heterogeneity and the lack of standardization in the methods used to extract and analyze stromal features. In this study, we employed Mueller matrix imaging and quantitative analysis to extract information-rich polarimetric parameters with tentative biophysical meaning to differentiate between two groups of patients with distinct local recurrence outcomes. Specifically, polarization signals based on D, t_1_, |m_43_|, q_L_, and $\upbeta$ metrics for patients who experienced local CRC relapse were significantly higher (and more broadly distributed) than for those who did not. These results suggest that PLM-measured CRC stromal features can provide useful prognostic information. However, for the potential clinical application of these promising initial results to be realized, further improvements are necessary. Future research will focus on exploring additional morphological polarimetric quantification methods to enhance the differentiation ability of MM parameters, utilizing deep neural networks, optimizing the pixels-ROIs-patients averaging pipeline, and investigating the possibility of automated ROI selection to minimize pathologists' subjectivity.

## Data Availability

Data underlying the results presented in this paper are not publicly available at this time but may be obtained upon reasonable request to K.T. (k.tumanova@mail.utoronto.ca).
